# Association of 2D and 3D transvaginal ultrasound findings with adenomyosis in symptomatic women of reproductive age: a prospective study

**DOI:** 10.6061/clinics/2021/e2981

**Published:** 2021-08-05

**Authors:** Ana Luiza Santos Marques, Marina Paula Andres, Leandro A. Mattos, Manoel O. Gonçalves, Edmund Chada Baracat, Mauricio Simões Abrão

**Affiliations:** I Departamento de Obstetricia e Ginecologia, Hospital das Clinicas HCFMUSP, Faculdade de Medicina, Universidade de Sao Paulo, Sao Paulo, SP, BR; II Divisao de Ginecologia, Hospital Beneficencia Portuguesa de Sao Paulo, Sao Paulo, SP, BR; III Departamento de Diagnostico por Imagem, Escola Paulista de Medicina da Universidade Federal de Sao Paulo (EPM-UNIFESP), Sao Paulo, SP, BR; IV Secao de Diagnosticos Pelvicos Femininos, Alta Excelencia Diagnostica, Sao Paulo, SP, BR

**Keywords:** Adenomyosis, Abnormal Uterine Bleeding, Dysmenorrhea, Pelvic Pain, Transvaginal Ultrasound

## Abstract

**OBJECTIVE:**

To evaluate the association of two-dimensional (2D) and three-dimensional (3D) transvaginal ultrasound (TVUS) findings with adenomyosis symptoms.

**METHODS:**

This prospective study conducted between January and December 2018 enrolled 78 women aged 18 to 40 years with abnormal uterine bleeding (AUB), infertility, and/or pelvic pain. All patients underwent 2D and 3D TVUS. Signs of adenomyosis on TVUS were identified according to the consensus of the Morphological Uterus Sonographic Assessment group.

**RESULTS:**

The prevalence of adenomyosis on TVUS was 55.12%. Patients with adenomyosis were older (*p*=0.002) and had more dysmenorrhea, AUB, and endometriosis than those without adenomyosis. When comparing the presence of symptoms with each adenomyosis feature, on 2D TVUS, severe dyspareunia was significantly associated with the presence of a poorly defined junctional zone (JZ) (*p*=0.023) and on 3D TVUS, patients with AUB had a more irregular (*p*=0.003), poorly defined (*p*=0.028), and interrupted JZ (*p*=0.011). After logistic regression analysis, signs of adenomyosis on TVUS remained significantly associated only with age over 30 years (OR: 1.2; 95% CI: 1.0-1.2) and AUB (OR: 7.65; 95% CI: 2-29). Patients with diffuse adenomyosis were older and presented with more infertility and AUB than patients with focal or no adenomyosis.

**CONCLUSION:**

The findings of adenomyosis by 2D and 3D TVUS showed association with age and AUB. 3D TVUS alterations in the JZ were associated with AUB and dyspareunia. Diffuse adenomyosis was associated with older age, a greater prevalence of infertility, and AUB.

## INTRODUCTION

Adenomyosis was once considered a disease of multiparous women in late reproductive age, usually diagnosed after hysterectomies (1). However, it has been gaining worldwide interest as it has become a multifaceted disease diagnosed by noninvasive imaging techniques in young women (2) with abnormal uterine bleeding (AUB), infertility, and pelvic pain and even in asymptomatic women (3). In addition, adenomyosis is often diagnosed in association with gynecological comorbidities such as endometriosis (4,5) and uterine fibroids (6).

Studies have shown that imaging tools such as magnetic resonance imaging and two-dimensional (2D) and three-dimensional (3D) transvaginal ultrasound (TVUS) can accurately diagnose women with diffuse adenomyosis with a sensitivity of 80-86% and 50-96% (7,8), respectively. However, few studies have investigated the early stages of adenomyosis, when patients are diagnosed with focal adenomyosis with mild or no symptoms (9-12). Therefore, clinical criteria and shared imaging criteria are still lacking, and data from previous studies are heterogeneous and not fully comparable (13). It is important to remember that there are controversies about pathogenic theories, classifications, and diagnostic imaging criteria that prevent a shared definition of adenomyosis, even after histopathological examination (14).

Although the diagnostic process of adenomyosis is currently challenging, this study, given the importance of TVUS as a first-line diagnostic examination in the patient’s gynecological investigation, aims to contribute by evaluating the association of 2D and 3D TVUS findings with adenomyosis symptoms.

## METHODS

### Study design

This prospective study was conducted at the Getúlio Vargas University Hospital (Federal University of Amazonas) in collaboration with the Endometriosis Section, Divisão de Clinica Ginecologica, Hospital das Clinicas Faculdade de Medicina, Universidade de São Paulo, São Paulo, Brazil. The study was approved by the Internal Review Board of both hospitals (IRB: 2.131.291/2017) and was conducted in accordance with the Helsinki Declaration. All participants provided written informed consent to participate in the study.

From January 2018 to December 2018, women aged 18-40 years with AUB, pelvic pain, and/or infertility, were recruited from the outpatient clinic of the Getúlio Vargas University Hospital. Patients who were pregnant, had cervical cancer, were in current use of hormonal treatment, had no previous vaginal intercourse, or had fibroids >8.0 cm (maximum diameter) or more than three fibroids > 5.0 cm (maximum diameter) were excluded (9). Infertility was defined as failure to achieve a clinical pregnancy after 12 months or more of regular unprotected sexual intercourse.

The clinical history of each patient was retrieved through an interview using a structured questionnaire by one researcher (ALSM). The questionnaire included age, height, weight, AUB, personal history of endometriosis or myoma, and history of infertility and pain (dysmenorrhea, dyspareunia, and chronic pelvic pain). For each painful symptom, the intensity was assessed using a visual analog scale from 0 to 10 (15).

**Figure 1:**
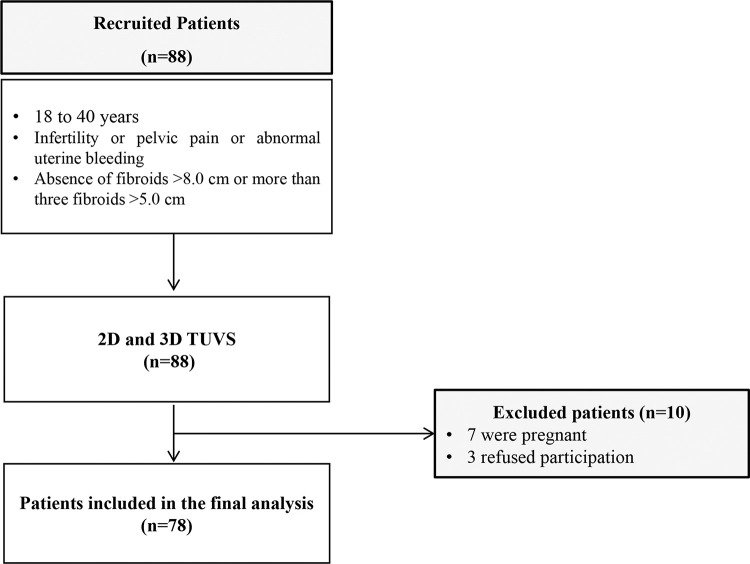
Flowchart of included patients.

All included women underwent 2D TVUS and 3D TVUS performed by an experienced sonographer (ALSM) with more than 18 years of experience in gynecological ultrasound. A total of 88 patients were eligible and underwent the TVUS protocol with bowel preparation (16). Of the 88 recruited patients, 10 were excluded from the study (seven for pregnancy and three for refusing participation), resulting in 78 patients included in the final analysis (Figure 1).

### Two- and three-dimensional transvaginal ultrasound

All TVUS examinations were performed using Voluson E8 Expert or Voluson E10 BT18 (GE Healthcare Ultrasound; Zipf, Austria) equipped with a multifrequency endovaginal probe (6-12 MHz) and a multifrequency 3D endovaginal probe (2.8-10 MHz) after bowel preparation.

The 2D TVUS evaluation included evaluation and measurement of the pelvic organs. The uterus and endometrium were measured, and the uterine volume was calculated using the ellipsoidal formula (longitudinal uterine diameter×transverse diameter×anteroposterior diameter×0.532). Any myometrial lesions were described and measured and if there were findings of pelvic endometriosis (ovaries, tubas, rectosigmoid, sigmoid, bladder, distal ureters, retrocervical region, rectovaginal septum and vagina), the extent of the disease was evaluated. Power Doppler was performed using preinstalled fixed settings (Frequency: 6-9 MHz; pulse repetition frequency: 0.6-0.3 kHz; gain: 4.0; wall motion filter: 40 Hz).

In the 3D TVUS evaluation, two to four uterus gray-scale static volumes were obtained from the sagittal and transverse planes. The volume acquisition technique was standardized, with frequency set to 6-9 MHz, uterus enlarged up to half the screen, sweep angle at 120°, scan speed adjusted from medium to maximum quality, and the 3D volume box exceeding uterine borders by 1 cm on each side. The rendering mode, or Omni View, was used for the reconstruction of the coronal plane; this consisted of placing a straight or curved line along the endometrial strip in the sagittal and transverse planes (Panels A and B in multiplanar mode). Multiplanar vision was then manipulated until a satisfactory coronal plane image of the uterine external profile and cavity was obtained, with bilateral visualization of the interstitial portion of the fallopian tube. The volume contrast image (slice thickness 2-4 mm) with volume rendering (mixed light surface and gradient light) was then applied. After acquisition, the ultrasound volumes were stored on the hard disk of the machine.

**Figure 2:**
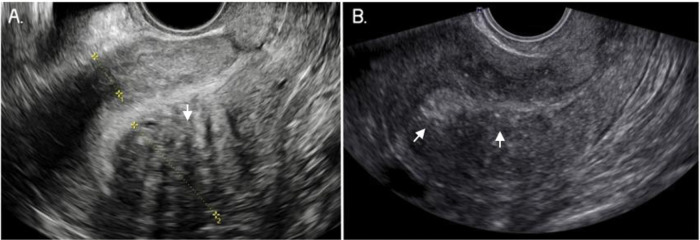
Two-dimensional ultrasound imaging of a uterus in a longitudinal section showing typical sonographic features of adenomyosis. **A.** Note the asymmetry of the myometrial wall (posterior wall thicker than anterior wall) and the heterogeneous myometrium with hypoechoic linear striae (arrows). **B.** Poorly defined junctional zone (arrows).

Adenomyosis was identified by TVUS using the consensus of the Morphological Uterus Sonographic Assessment group (10). The 2D TVUS features were a heterogeneous uterus, myometrial cysts, hypoechoic linear striae (when a radiating pattern of thin acoustic shadows not arising from echogenic foci or leiomyoma, fan-shaped shadowing was present), hyperechogenic islands (buds), a poorly defined junctional zone (JZ), and posterior and anterior wall asymmetry. Uterine wall asymmetry was assessed by measuring in the sagittal plane, perpendicular to the endometrium, from the uterine serosa external to the external endometrial contour; measurements included the JZ but not the endometrium. Both measurements were recorded from the same image and ideally obtained from the thickest point of the myometrial wall. The ratio between the anterior and posterior wall thickness was calculated. A ratio of approximately 1 indicates that the myometrial walls are symmetrical and a ratio well above or below 1 indicates asymmetry, although this may also be estimated subjectively (Figure 2). Power Doppler was used in 2D mode to differentiate adenomyomas from leiomyomas and myometrial cysts from gaps or vascular components. The vascular pattern within the myometrium can be uniform or non-uniform, and the vascular pattern of a myometrial lesion can be circumferential, translesional, or both. The type of translesional vascularization, which is characterized by the presence of vessels perpendicular to the uterine/serous cavity crossing the lesion, is more frequent in adenomyoma, while the circumferential type is typical of fibroids (10,17).

**Figure 3:**
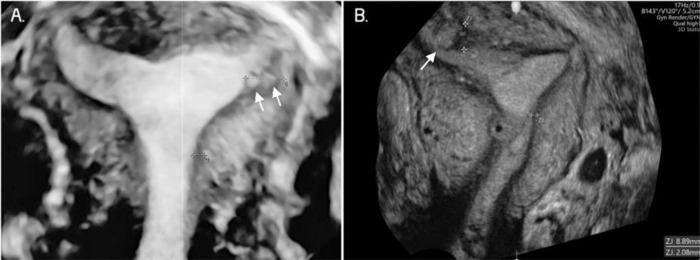
Three-dimensional ultrasound imaging of a uterus with adenomyosis in the coronal plane. **A.** Protrusions of the endometrium into the junctional zone (JZ) (arrows). **B**. Junctional zone thickened, interrupted, and infiltrated by subendometrial echogenic nodules (buds) (arrows). In this image, the JZ difference is 6.8 mm.

**Figure 4:**
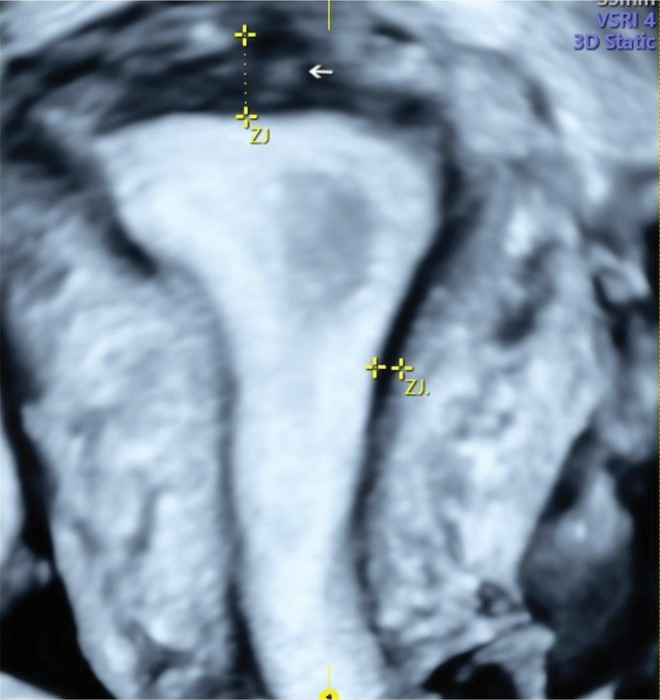
Three-dimensional ultrasound imaging of a uterus in the coronal plane showing focal adenomyosis of the inner myometrium: junctional zone interrupted with one hyperechogenic island (arrows).

**Figure 5:**
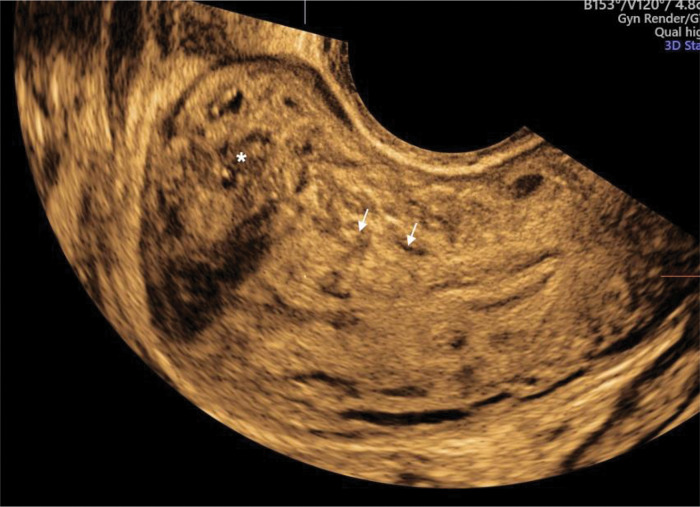
Two-dimensional ultrasound imaging of a uterus in the sagittal plane showing a focal adenomyosis of the outer myometrium: a heterogeneous uterus with adenomyoma and an irregular junctional zone (arrows and asterisks).

**Figure 6:**
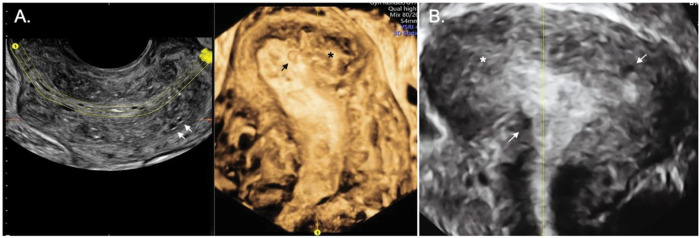
Two and three-dimensional ultrasound imaging of a uterus with diffuse type adenomyosis. **A.** Heterogeneous uterus with an irregular and interrupted junctional zone associated with myometrial cysts (arrows). **B.** Severe diffuse adenomyosis. Globular, enlarged, and heterogeneous uterus with myometrial cysts and an ill-defined junctional zone, completely infiltrated by adenomyosis (arrows and asterisks).

The 3D TVUS (Figure 3) evaluated the characteristics of the JZ. If it was found to be poorly defined, irregular, and interrupted and if its thickness was ≥8 mm or the difference between the maximum and minimum thickness was ≥4 mm (JZ Dif) (17), it was considered suggestive of adenomyosis. We considered focal and diffuse adenomyosis of the outer and inner myometrium separately and classified adenomyosis types according to TVUS features as assessed in the coronal plane. The focal of the inner myometrium, or JZ type, was a JZ interrupted with one hyperechogenic island (bud, Figure 4). The focal of the outer myometrium type was a heterogeneous uterus with irregular JZ and hyperechogenic subendometrial lines or buds, heterogeneous uterus with one cystic area within the myometrium, or heterogeneous uterus with adenomyoma (Figure 5). Diffuse type was defined by three or more findings evaluated by 2D-TVUS or 3D-TVUS or the presence of a question mark sign (18) (Figure 6). The “Question Mark Sign” was defined when the uterus was flexed backward, with the uterus fundus facing the posterior pelvic compartment with the cervix directed frontally toward the urinary bladder (19).

### Statistical analysis

For the comparison of the means, Student’s t-test or analysis of variance was used; however, for the comparison of frequencies, the nonparametric Fisher’s exact test or chi-square test with Yates correction was used. For the comparison of TVUS characteristics and clinical symptoms, the latter were considered as independent variables and the former as dependent variables. Multivariate analysis was performed using a logistic regression analysis. Variables with *p*<0.02 were included in the model.

A significance level of 5% was considered statistically significant. Data were analyzed using the Epi Info software (version 7.2 for Windows program; US Centers for Disease Prevention and Control, www.cdc.gov/epiinfo) and the SPSS (v25) software.

## RESULTS

Of the 78 patients included in the study, findings of adenomyosis were observed by 2D TVUS only in 1 patient, by 3D TVUS only in 2 patients, and by both 2D and 3D TVUS in 40 (55.1%) patients, resulting in 43 patients with any features of adenomyosis. Adenomyosis was classified as focal of the inner myometrium in 22 (51.1%) cases, focal of the outer myometrium in 11 (25.6%) cases, and diffuse in 10 (23.3%) cases. Fibroids were present in 11.4% (4 out of 35) of patients without adenomyosis and in 30.2% (13 out of 43) of patients with adenomyosis.

The mean age of patients with TVUS findings of adenomyosis was greater than that of patients without TVUS findings of adenomyosis (31.6±5.8 years *versus* 27.4±5.7 years; *p*=0.002; Table 1). Patients with any sonographic finding of adenomyosis presented with significantly more AUB (23.3% *vs*. 0%; *p*=0.002) and significantly more dysmenorrhea (97.7% *versu*s 82.9%; *p*=0.041; Table 1) than those without any sonographic finding of adenomyosis. 2D TVUS findings of adenomyosis were significantly associated with endometriosis (55.8% *versus* 25.7%; *p*=0.011, Table 1), with 45% deeply infiltrating endometriosis of the posterior compartment and 10% ovarian endometriomas associated with posterior compartment endometriosis. We did not find any endometriosis in the anterior compartment.

The most frequently observed ultrasonographic features of adenomyosis by 2D TVUS are summarized in Table 2 and included a heterogeneous myometrium (n=36; 83.7%), subendometrial echogenic nodules (n=27; 62.8%), hypoechoic linear striae (n=19; 44.2%), and a globular uterus (n=17, 39.5%). The most frequent 3D TVUS features observed were interrupted JZ (n=38; 88.4%) and JZ thickness ≥4 mm (n=22; 56.4%).

### Comparison of clinical characteristics with TVUS findings

When comparing the presence of symptoms with each adenomyosis feature on 2D TVUS, patients with severe dyspareunia had more poorly defined JZ (81.3% *vs*. 56.5%; *p*=0.023; Table 3) than those without severe dyspareunia. When comparing symptoms with each adenomyosis feature on 3D TVUS, patients with AUB showed more irregular JZ (86.5% *vs*. 56.1%; *p*=0.003), more poorly defined JZ (100% *versus* 65.7%; *p*=0.028), and more interrupted JZ (77.8% *versus* 57.5%; *p*=0.011) than those without AUB. After logistic regression analysis, the presence of sonographic signs of adenomyosis remained significantly associated only with age over 30 years (OR: 1.2; 95% CI: 1.0-1.2; *p*=0.0375; Table 3) and AUB (OR: 7.65; 95% CI: 2-29; *p*=0.0029; Table 4).

When comparing the type of adenomyosis, patients with diffuse adenomyosis were older (36.7±2.6 years) than patients in all other groups (focal adenomyosis of the inner myometrium, 30.5±5.6 years; focal adenomyosis of the outer myometrium, 29.4±5.7 years; without adenomyosis, 27.4±5.7 years; *p*<0.001; Table 5). In addition, all patients with diffuse adenomyosis presented with infertility and AUB, and these frequencies were significantly higher than those in patients with focal or without adenomyosis (Table 5).

## DISCUSSION

The aim of this study was to evaluate the association of adenomyosis symptoms (infertility, pelvic pain, and AUB) with 2D and 3D TVUS findings in young women of reproductive age. Although 2D and 3D TVUS features have been shown to be associated with adenomyosis (14), the association of each feature or its combination with clinical symptoms such as pain, bleeding, and pregnancy needs to be defined.

In this study, in 55.8% of cases, an association between adenomyosis and endometriosis was observed, as assessed by TVUS with bowel preparation. Both diseases share common symptoms such as dysmenorrhea, infertility, dyspareunia, and chronic pelvic pain and are reported to be associated in 13.2-89.4% of cases (20-23). It was also previously reported (18) that women with diffuse adenomyosis showed a higher association with endometriosis than those with mild adenomyosis.

One study (23) evaluated the TVUS findings of adenomyosis and clinical symptoms in 718 patients. The researchers observed that 2D TVUS features of adenomyosis were present in 21.9% of symptomatic women requiring consultation and that they were significantly associated with the severity of menstrual pain. In our study, which included patients with infertility, AUB, and/or pelvic pain, 55% of women presented with sonographic signs of adenomyosis. Moreover, we observed that patients with severe dysmenorrhea and AUB had a higher prevalence of TVUS features of adenomyosis than those without these symptoms (97.7% *versus* 82.9%; 23.3% *versus* 0%, respectively). This is in accordance with a previous study (18), which showed a prevalence of 72.2% of TVUS signs in patients with severe dysmenorrhea.

The authors previously reported that when adenomyosis is detected late, a greater number of ultrasound features are observed, and patients present with worse symptoms and more severe disease. One study (23) showed that the greater the number of adenomyosis features identified on 2D TVUS, the greater the pain score. Another similar study (2) also reported a significant association between the number of 2D TVUS adenomyosis features with dysmenorrhea intensity and heavy menstrual bleeding in a cohort of nulliparous women, aged 18-30 years.

A recent study (18) included 108 patients with sonographic signs of adenomyosis. The study reported that patients with older age and heavy menstrual bleeding were more associated with diffuse adenomyosis than those with focal disease with no difference in the presence of dysmenorrhea and dyspareunia. The study (18) also showed that in patients trying to conceive, the presence of ultrasound findings of focal disease was associated with a higher percentage of infertility (82% *versus* 52%; *p*<0.005) and miscarriage (69% *versus* 36%; *p*<0.05) than that of diffuse disease. Similarly, in our study, after logistic regression analysis, clinical variables associated with the presence of adenomyosis were age >30 years and AUB. In contrast, we observed a higher frequency of infertility in patients with diffuse adenomyosis (100%) than in those with focal adenomyosis of the inner (72.7%) and outer (81.8%; *p*<0.001) myometrium.

The limitations of this study include the small number of patients and the absence of histological analysis to evaluate the accuracy of TVUS as we included only women with reproductive desire. The main strengths of our study were the inclusion criteria, the prospective design, and the fact that all TVUS were performed by only one ultrasonographer, minimizing interobserver variability.

## CONCLUSION

Findings of adenomyosis by 2D and 3D TVUS were associated with age and AUB. 3D TVUS alterations of the JZ were associated with AUB and dyspareunia. Diffuse adenomyosis was associated with older age, greater prevalence of infertility, and AUB.

## AUTHOR CONTRIBUTIONS

Marques ALS was responsible for the study design, collection of data, analysis of results and manuscript drafting. Andres MP and Abrão MS were responsible for the study design, analysis of results and manuscript drafting. Mattos LA and Gonçalves MO were responsible for the study design and collection of data. Baracat EC was responsible for the study design and manuscript drafting.

## Figures and Tables

**Table 1:** Characteristics of included patients evaluated by TVUS.

Characteristics	With TVUS signs of adenomyosis (n=43)		Without TVUS signs of adenomyosis (n=35)		Total	*p*
	n	%	n	%		
Age	31.6±5.8		27.4±5.7			**0.002^a^**
BMI (kg/m^2^)	25.7±4.0		24.2±5.2			0.158^a^
Previous pregnancy	3	7.0	0	0	3	0.248^b^
AUB	10	23.3	0	0	10	**0.002^b^**
Severe Dysmenorrhea (VAS ≥ 7)	42	97.7	29	82.9	71	**0.041^b^**
Severe Dyspareunia (VAS ≥ 7)	33	76.7	21	60.0	54	0.178^c^
Severe Acyclic pain (VAS ≥ 7)	32	74.4	20	57.1	52	0.171^c^
Myoma	13	30.2	4	11.4	17	0.084^c^
Endometriosis	24	55.8	9	25.7	33	**0.011^c^**

TVUS: transvaginal ultrasound; BMI: body mass index; AUB: abnormal uterine bleeding; VAS: visual analog scale of pain; ^a^Student’s t-test; ^b^Fisher’s exact test; ^c^Chi-square test with Yates correction. P-values in bold indicate statistical differences at the 5% significance level.

**Table 2:** Prevalence of 2D and 3D transvaginal ultrasound features in patients with adenomyosis.

Adenomyosis findings	n	*%*
2D TVUS	42	55.0
Heterogeneous myometrium	36	83.7
Buds	27	62.8
Linear striae	19	44.2
Globular uterus	17	39.5
Uterine wall asymmetry	15	34.9
Myometrial cysts	14	32.6
Poorly defined JZ	5	11.6
Question Mark sign	4	9.3
3D TVUS	41	
Interrupted JZ	38	88.4
JZ Dif (≥4.0 mm)	22	56.4
Irregular JZ	21	48.8
Defined JZ	3	7.0
Poorly defined JZ	4	9.3

2D: two dimensions; 3D: three dimensions; TVUS: transvaginal ultrasound; JZ: junctional zone; JZ Dif: difference between the maximum and minimum thickness of the junctional zone.

**Table 3:** Comparison of the presence of clinical symptoms and TVUS signs of adenomyosis.

Characteristic	Infertility	Severe Dyspareunia (VAS≥7)	Severe Dysmenorrhea (VAS≥7)	Severe Acyclic pain (VAS≥7)	AUB
2D TVUS					
Globular uterus					
No	19 (79.2)	27 (64.3)	28 (66.7)	26 (61.9)	30 (71.4)
Yes	28 (90.3)	25 (69.4)	20 (55.6)	24 (66.7)	25 (69.4)
*p*	0.276^a^	0.630^b^	0.315^b^	0.662^b^	0.848^b^
Asymmetry					
No	22 (75.9)	33 (66.0)	29 (58.0)	32 (64.0)	34 (68.0)
Yes	25 (96.2)	19 (67.9)	19 (67.9)	18 (64.3)	21 (75.0)
*p*	0.054^a^	0.867^b^	0.391^b^	0.980^b^	0.515^b^
Myometrial cysts					
No	36 (81.8)	44 (66.7)	40 (60.6)	43 (65.2)	45 (68.2)
Yes	11 (100)	8 (66.7)	8 (66.7)	7 (58.3)	10 (83.3)
*p*	0.188^a^	0.999^a^	0.758^a^	0.747^a^	0.492^a^
Buds					
No	42 (85.7)	48 (66.7)	45 (62.5)	45 (62.5)	49 (68.1)
Yes	5 (83.3)	4 (66.7)	3 (50.0)	5 (83.3)	6 (100)
*p*	0.999^a^	0.999^a^	0.670^a^	0.411^a^	0.172^a^
Linear striae					
No	39 (83.0)	46 (65.7)	41 (58.6)	45 (64.3)	48 (68.6)
Yes	8 (100)	6 (75)	7 (87.5)	5 (62.5)	7 (87.5)
*p*	0.587^a^	0.712^a^	0.143^a^	0.999^a^	0.424^a^
Poorly defined JZ					
No	20 (83.3)	26 (56.5)	26 (56.5)	27 (58.7)	29 (63.0)
Yes	27 (87.1)	26 (81.3)	22 (68.8)	23 (71.9)	26 (81.3)
*p*	0.718^a^	**0.023^b^**	0.275^b^	0.233^b^	0.083^b^
**3D TVUS**					
Irregular JZ					
No	17 (89.5)	24 (58.5)	24 (58.5)	26 (63.4)	23 (56.1)
Yes	30 (83.3)	28 (75.7)	24 (64.9)	24 (64.9)	32 (86.5)
*p*	0.700^a^	0.109^b^	0.566^b^	0.894^b^	**0.003^b^**
Poorly defined JZ					
No	38 (86.4)	45 (67.2)	42 (62.7)	44 (65.7)	44 (65.7)
Yes	9 (81.8)	7 (63.6)	6 (54.5)	6 (54.5)	11 (100)
*p*	0.654^a^	0.999^a^	0.741^a^	0.511^a^	**0.028^a^**
Interrupted JZ					
No	16 (88.9)	23 (57.5)	23 (57.5)	25 (62.5)	23 (57.5)
Yes	22 (84.6)	22 (81.5)	19 (70.4)	19 (70.4)	21 (77.8)
*p*	0.890^a^	0.127^a^	0.557^a^	0.610^a^	**0.011^a^**

2D: two dimensions; 3D: three dimensions; TVUS: transvaginal ultrasound; AUB: abnormal uterine bleeding; VAS: visual analog scale for pain; ^a^Fisher’s exact test; ^b^Chi-square test. Significant differences are highlighted in boldface.

**Table 4:** Comparison of clinical symptoms in patients with TVUS findings of adenomyosis.

Characteristics	OR	CI 95%	*p* ^a^
Age >30 years	1.12	1.01-1.24	**0.0375**
Endometriosis	2.28	0.65-7.95	0.1966
Severe dysmenorrhea (VAS≥7)	9.48	0.52-170.64	0.1274
Severe dyspareunia (VAS≥7)	0.67	0.17-2.66	0.5744
Leiomyomas	6.86	0.99-47.46	0.0511
Severe acyclic pain (VAS≥7)	2.37	0.61-9.23	0.2128
AUB	7.64	2.01-29.09	**0.0029**

TVUS: transvaginal ultrasound; VAS: visual analog scale; OR: odds ratio; CI 95%: confidence interval 95%; ^a^Logistic regression. Significant differences are highlighted in boldface.

**Table 5:** Comparison of adenomyosis severity and symptoms.

Characteristic	Adenomyosis				*p*
	No	Focal (inner myometrium)	Focal (outer myometrium)	Diffuse	
N	35	22	11	10	
Age	27. 4±5.7	30.5±5.6	29.4±5.7	36.7±2.6	**<0.001^a^**
Infertility	14 (40.0)	16 (72.7)	9 (81.8)	10 (100)	**<0.001^b^**
Severe Dyspareunia (VAS≥7)	17 (48.6)	14 (63.6)	8 (72.7)	8 (80.0)	0.21^b^
Severe Dysmenorrhea (VAS≥7)	29 (82.9)	22 (100)	10 (90.9)	10 (100)	0.11^b^
Severe Acyclic pain (VAS≥7)	20 (57.1)	15 (68.2)	8 (72.7)	8 (80.0)	0.506^b^
AUB	16 (45.7)	18 (81.8)	8 (72.7)	10 (100)	**0.002^b^**

AUB: abnormal uterine bleeding; VAS: visual analog scale; ^a^ANOVA: analysis of variance.

^b^Chi-square test. Significant differences are highlighted in boldface.
